# Red and Blue Netting Alters Leaf Morphological and Physiological Characteristics in Apple Trees

**DOI:** 10.3390/plants10010127

**Published:** 2021-01-09

**Authors:** Richard M. Bastías, Pasquale Losciale, Camilla Chieco, Luca Corelli-Grappadelli

**Affiliations:** 1 Departamento de Producción Vegetal, Facultad de Agronomía, Universidad de Concepción, 3780000 Chillán, Chile; 2 Department of Soil, Plant and Food Sciences, University of Bari “Aldo Moro”, 70121 Bari, Italy; pasquale.losciale@uniba.it; 3 Institute of BioEconomy, National Research Council of Italy, 40129 Bologna, Italy; camilla.chieco@ibe.cnr.it; 4 Department of Agricultural and Food Sciences, University of Bologna, 40126 Bologna, Italy; luca.corelli@unibo.it

**Keywords:** photo-selective nets, light quantity and quality, leaf mesophyll, leaf stomata, leaf gas exchanges

## Abstract

There is little information about the role of red and blue light on leaf morphology and physiology in fruit trees, and more studies have been developed in herbaceous plants grown under controlled light conditions. The objective of this research was to evaluate the effect of red and blue screens on morpho-anatomy and gas exchange in apple leaves grown under ambient sunlight conditions. Apple trees cv. Fuji were covered by 40% red and blue nets, leaving trees with 20% white net as control. Light relations (photosynthetic photon flux density, PPFD; red to far-red light ratio, R/FR and blue to red light ratio, B/R), morpho-anatomical features of the leaf (palisade to spongy mesophyll ratio, P/S, and stomata density, SD) and leaf gas exchange (net photosynthesis rate, A_n_; stomatal conductance, *g*_s_; transpiration rate, *E*; and intrinsic water use efficiency, IWUE) were evaluated. Red and blue nets reduced 27% PPFD, reducing by 20% SD and 25% P/S compared to control, but without negative effects on A_n_ and *g*_s_. Blue net increased *g*_s_ 21%, leading to the highest *E* and lowest IWUE by increment of B/R light proportion. These findings demonstrate the potential use of red and blue nets for differential modulation of apple leaf gas exchange through sunlight management under field conditions.

## 1. Introduction

Netting is a cultural practice in apple orchards to ensure sustainable fruit production and quality. Nets are used to protect apple fruit and plant from hail damage in areas with high frequency of hailstorms [1], solar injury in regions with excessive radiation and higher temperatures [2,3,4], as well to protect the orchard from damage by insects [5]. Covering apple trees with nets alters orchard microclimate, with changes in the incoming solar radiation, air temperature, relative humidity, wind speed and soil moisture with ensuing effects on physiological plant and fruit responses [3,6]. The use of photo-selective colored nets demonstrates the interest in modifying the solar light quality conditions to promote desired plant physiological responses, improving the yield and fruit quality in different horticultural crops [7]. Colored nets have been tested under field conditions with different effects on productivity and fruit quality traits, depending on the net color and fruit crops. In blueberry, red and white nets provide a harvest delay without detrimental effects on return bloom, yield and fruit quality [8]. In kiwifruit, red and white nets allowed a high dry matter accumulation in the fruit, while a reduction of fruit dry matter accumulation was observed in kiwifruit grown under blue and grey nets [9]. In citrus, pearl and yellow colored nets increased the root and shoot development, improving plant growth performance under semi-arid conditions [10]. In ‘Golden Delicious’ apple, yield was significantly increased by pearl and red nets [7]. In ‘Fuji’ apple, blue and gray nets increased significantly fruit growth compared to red net [11], while fruit quality traits such as color, sweetness, hardness and fruit peel antioxidant content were also affected by colored nets [12,13].

From a physiological point of view the effect of colored nets has been more variable, depending on net color, crops and cultivars. In blueberry, the net color has a weaker effect on leaf photosynthesis compared to its shading effect: the reduction of net photosynthesis was more closely related to the decrease in available photosynthetic photon flux density (PPFD) than spectra light conditions generated by red and white nets [14]. In citrus, the use of aluminized net improved leaf net photosynthesis, which was primarily attributed to reduced photo-inhibition by the decrease in the excessive solar light under the net [15]. In ‘Royal Gala’ apple, the reduction of photosynthetic photon flux density by black net negatively affected photosynthesis and stomatal conductance in leaves, suggesting that leaf anatomical modifications induced by low light availability under the net were limiting photosynthetic capacity [2]. In ‘Fuji’ apple, the leaf photosynthesis rate was not affected by colored nets [1], but the use of blue net increased the leaf photosynthesis compared to red net [11]. In ‘Honeycrisp’ apple grown under a high light and arid environment, the use of blue net increased the leaf-level photosynthetic light-use efficiency, reducing leaf photo-inhibition, but without significant effects on leaf photosynthesis [16]. These inconsistent results suggest the need to study in more detail the specific mechanism(s) involved in the effect of net color on the tree physiological responses [3].

It has been demonstrated that colored nets affect light quality transmission with significant changes of the blue (400–500 nm) and red (600–700 nm) light proportion, which alters different plant physiological and morphological responses mediated by the photoreceptors phytochrome, cryptochrome and phototropins [1,17]. Different leaf traits affecting photosynthesis are altered by the red and blue light [18], including anatomical changes in the palisade and spongy mesophyll. Combination of blue with far-red light reduced significantly the thickness of mesophyll palisade layers compared to red combined with far-red light [19], while thickness was increased when red light was supplemented by blue [20]. Light quality also affects leaf chlorophyll content. Supplemental blue light enhanced the enzymatic biosynthesis of chlorophyll, while under red light chlorophyll synthesis was diminished [21]. The influence of blue and red light on stomatal opening has been also documented: blue promotes stomata opening by direct hyperpolarization of the membrane potential, while red light induces stomatal opening by driving photosynthesis and thus decreasing intercellular CO_2_ concentration stimulates stomatal opening and allowing CO_2_ increase [22].

Although different experiments have demonstrated the role of red and blue light in the regulation of photosynthesis, most of these studies have been carried out with herbaceous plants and under controlled light conditions, and poor information exists about how red and blue nets affect these processes in apple trees grown under solar light. The aim of this study was to evaluate the effect of red and blue shading nets on apple trees grown under solar light conditions on the following aspects: (1) changes in light quantity and quality; (2) leaf morphological and anatomical parameters and (3) leaf gas exchange parameters.

## 2. Results

### 2.1. Light Relations

**Figure 1:**
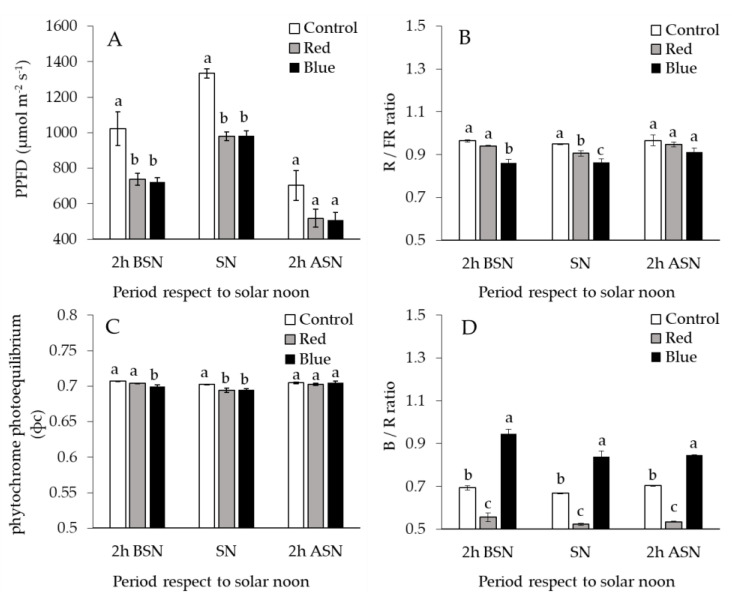
(**A**) The influence of red and blue netting on total photosynthetic photon flux density, PPFD, (**B**) red to far-red light ratio, R/FR, (**C**) phytochrome photoequilibrium, Ф_c_, (**D**) blue to red light ratio, B/R under solar ambient conditions; 2 h BSN: Two hours before of solar noon; SN: solar noon; 2 h ASN: Two hours after solar noon. Columns with different letters are statistically significant by the LSD Fischer test; *n* = 4.

The incident PPFD did not differ significantly among red and blue nets when measured at different times during the day. On average, PPFD under red and blue nets was reduced by 27% compared to control net (Figure 1A). Red to far-red light ratio (R/FR) values under blue and red nets were 10% and 5% lower than control net, respectively. These differences were statistically significant 2 h before solar noon for blue net and at solar noon for both colored nets (Figure 1B). Phytochrome photoequilibrium (ф_c_) value was also significantly reduced by colored nets (Figure 1C). The ф_c_ value was 1% lower than control net 2 h before solar noon for blue net and at solar noon for both colored nets (Figure 1C). Blue net increased significantly the daily blue to red light (B/R) ratio (27%) compared to control net, while red net reduced significantly this proportion (−22%) compared with the control net (Figure 1D).

**Figure 2:**
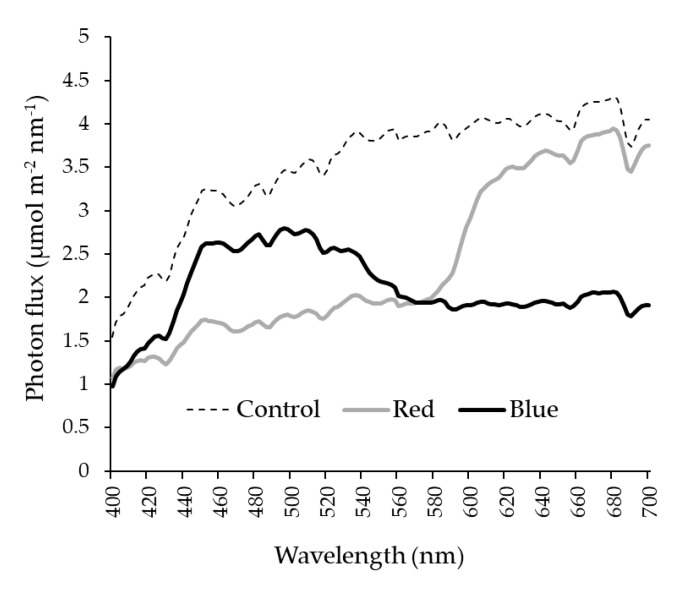
Spectral irradiance pattern measured in apple trees grown under blue, red and white control nets.

The amount of blue radiation (400–500 nm) under the blue net was on average 0.6 µmol m^−2^ s^−1^ nm^−1^ greater than under the red net (Figure 2). Blue net reduced on average 1.6 µmol m^−2^ s^−1^ nm^−1^ the radiation transmission in the red spectrum (600–700 nm) compared to red net (Figure 2).

### 2.2. Trees and Leaf Characteristics

Color nets seemed not to affect the leaf area (LA) and the trunk cross section area (TCSA) respect to the control. Conversely, blue net reduced the leaf mass area (LMA) by 8%, while Red net did not affect the LMA values in apple trees. Stomatal density was significantly lower on leaves developed under blue and red nets. Leaves on trees grown under color nets showed a 17% reduction in the number of stomata per mm^2^ respect to control net (Table 1). Chlorophyll content of leaves that grew under blue net was significantly higher (5%) than those under red and control nets. Finally, total shoot length (TSL) of trees under blue net was 70% higher than those under red and control nets (Table 1). Tree vigor did not significantly affect the leaf morphology and the chlorophyll content, just as it did not influence the trees size (Table 1).

**Figure 3:**
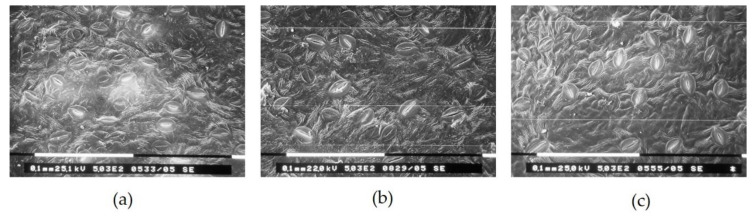
(**a**) Scanning electron micrograph of stomata characteristics in apple leaves under white (control), (**b**) red and (**c**) blue colored nets. Magnification 503×. White Bars = 100 µm.

Scanning electron microscopy (SEM) analysis confirmed that some stomatal characteristics were affected by color nets (Figure 3). Stomata length (SL) of leaves grown under Red net was significantly greater (11%) than those grown under control net, whereas there were no significant differences in SL among red and blue nets (Table 2). The ratio length/width (L/W) of the stomata was significantly affected by colored nets. Leaves grown under red and blue nets presented an increment of 7% in the L/W stomata compared to control net (Figure 3b,c; Table 2). Stomata frequency by length was also significantly affected by colored nets. The proportion of longest stomata (20–25 µm) in leaves grown under blue and red nets was 10–15% higher than control net (Table 2).

**Figure 4:**
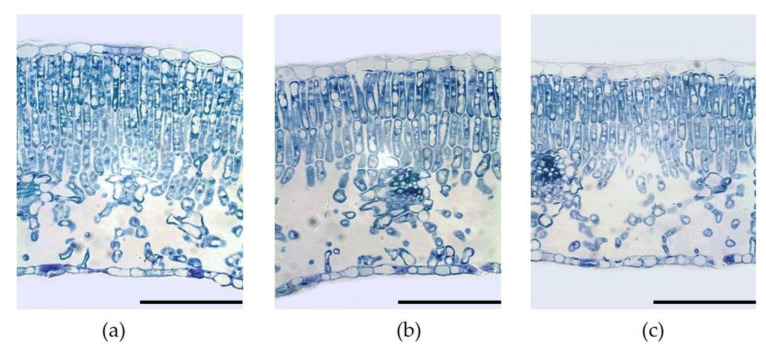
(**a**) Leaf cross-section of ‘Fuji’ mature apple leaves grown under white (control), (**b**) red and (**c**) blue nets. Magnification 40×. Bars = 100 µm.

Leaves under control net were thicker (228 µm) than leaves grown under red and blue nets (218–215 µm), but these differences were not significant (Figure 4; Table 3). Only palisade tissue seemed to be affected by colored nets. The palisade parenchyma of leaves grown under control net was 19% thicker than that of leaves grown under red and blue nets. Furthermore, leaves under control net showed three well defined layers of palisade cells (Figure 4a) while the palisade tissue observed in leaves from red and blue nets presented only two well defined layers of cells (Figure 4b,c). Although palisade thickness did not differ significantly among red and blue nets (Table 3), the ratio palisade/spongy mesophyll was significantly affected by colored nets. Leaves grown under red and blue nets presented a reduction of 25% in the ratio palisade/spongy mesophyll compared to control net (Figure 4b,c; Table 3). Leaf upper and lower epidermis thickness did not differ among colored nets (Table 3).

### 2.3. Leaf Gas Exchange

Evaluation of leaf gas exchange under controlled light conditions showed that red and blue nets had a significant effect on leaf net photosynthesis rate (A_n_), stomatal conductance (*g*_s_) and transpiration rate (*E*) (*p* < 0.01 and 0.05), while the leaf gas exchange parameters were not significantly affected by tree vigor or by the interaction between tree vigor and colored nets (Table 4). A_n_ was increased 30% and 15% by blue and red nets compared to control net, respectively. On average, *g*_s_ and *E* were incremented 44% and 30% by both colored nets, respectively, compared to control net (Table 4).

**Figure 5:**
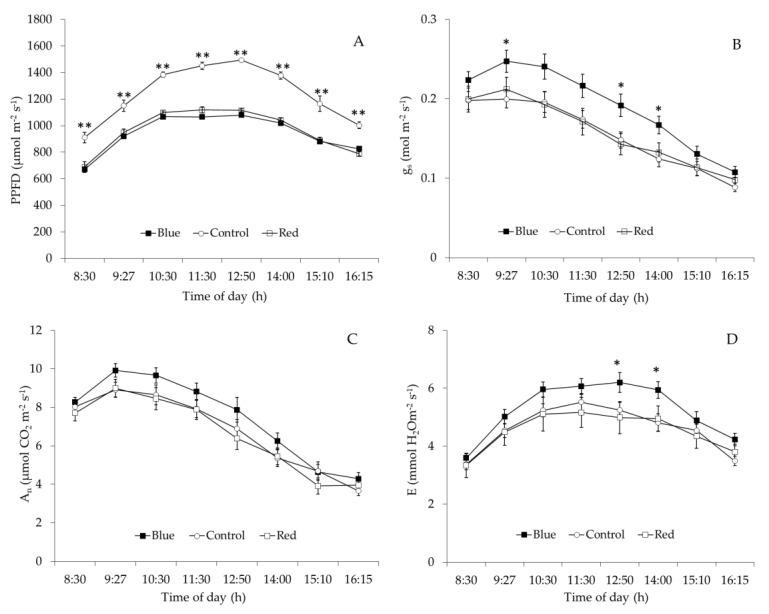
(**A**) Daily course of photosynthetic photon flux density, PPFD, (**B**) stomatal conductance, *g*_s_, (**C**) photosynthesis rate, A_n_ and transpiration rate, E (**D**) in ‘Fuji’ apple leaves grown under red (□), blue (■) and white control (○) nets. Each value represents the mean ± SE of 12 leaves measured under ambient light conditions on two summer days. *; **: significant and highly significant at *p* < 0.05 and 0.01, respectively.

Red and blue nets reduced significantly (*p* < 0.01) and in the same proportion the daily course of PPFD incident on the leaf and compared to control net (Figure 5A). Before solar noon (8:30–10:30 h), the incoming PPFD on the leaves grown under Blue and Red nets was on average 900 µmol m^−2^ s^−1^, representing a reduction of 22% of PPFD compared to control net (1150 µmol m^−2^ s^−1^). Near solar noon (11:30–12:50 h), the incoming PPFD under blue and red nets was on average 1100 µmol m^−2^ s^−1^, indicating a reduction of 26% of PPFD related to control (1500 µmol m^−2^ s^−1^). After solar noon (14:00–16:15 h), the PPFD under blue and red nets was on average 910 µmol m^−2^ s^−1^, representing a reduction of 23% of PPFD compared to control net (1180 µmol m^−2^ s^−1^). Over the whole day, blue net increased 21% the *g*_s_ compared with red and control nets and these differences were significantly largest (30%) at 12:50 and 14:00 h (Figure 5B). The positive effect of blue net on leaf *g*_s_ was also linked to increment in plant transpiration (Figure 5B,D). Maximum and significant values (*p* < 0.05) of leaf E under blue net were reached at 12:50 h and 14:00 h; these values of leaf E were on average 21% higher than those measured under red and control nets (Figure 5D). Although daily leaf A_n_ pattern was similar to *g*_s_, no differences were found in net CO_2_ assimilation (Figure 5C).

**Figure 6:**
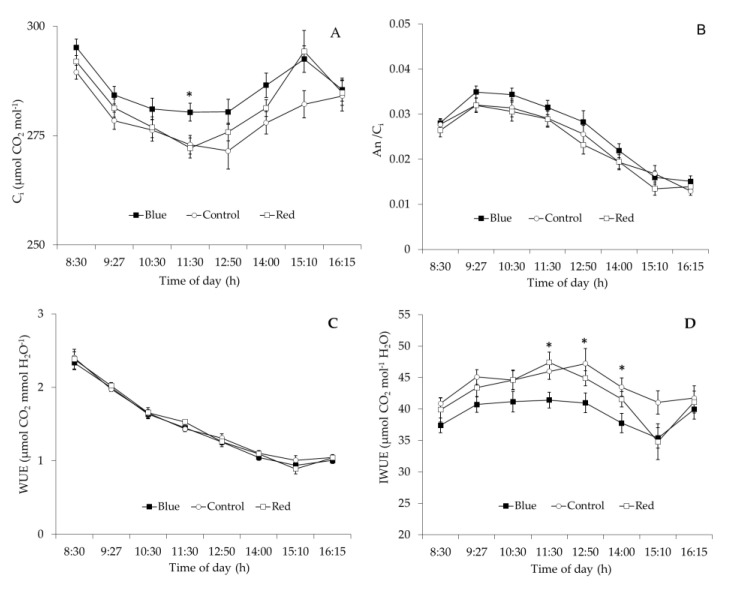
(**A**) Daily course of intercellular CO_2_ concentration, C_i_; (**B**); instantaneous carboxylation efficiency, A_n_/C_i_; (**C**) water use efficiency, WUE; and intrinsic water use efficiency, IWUE (**D**) in ‘Fuji’ apple leaves grown under red (□), blue (■) and white control (○) nets. Each value represents the mean ± SE of 12 leaves measured under ambient light conditions on two summer days. *: Significant at *p* < 0.05.

**Figure 7:**
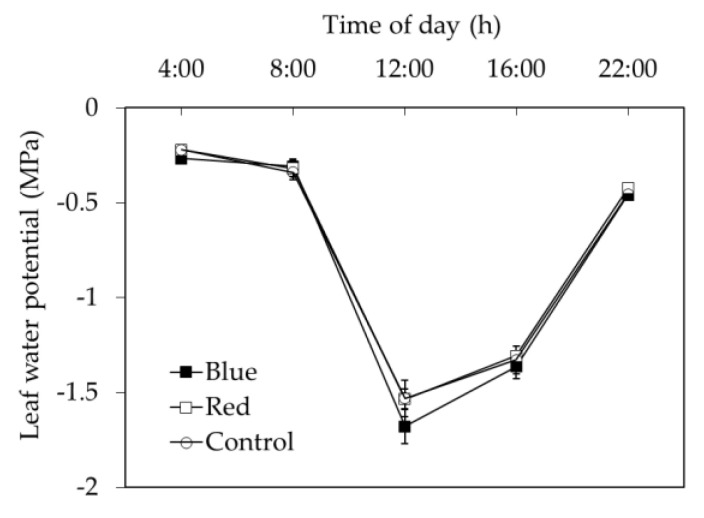
Daily course of leaf water potential in ‘Fuji’ apple potted trees grown under blue (■), red (□), and white control (○) nets. Each value represents the mean ± standard error (SE) of 5–10 leaves.

Blue net increased 2% the C_i_ compared with red and control nets and these differences were significant (*p* < 0.05) at 11:30 h (Figure 6A). Although the daily value of A_n_/Ci and water use efficiency (WUE) did not differ among nets, leaf intrinsic water use efficiency (IWUE) values under blue net were significantly (*p* < 0.05) lower than red net and control at 11:30, 12:50 and 14:00 h. (Figure 6B–D). Between 11:30 and 14:00 h, leaf IWUE under blue net decreased 11%, compared to that measured in leaves grown under red and control nets (Figure 6D), while daily stem water potential did not differ among net treatments (Figure 7).

**Figure 8:**
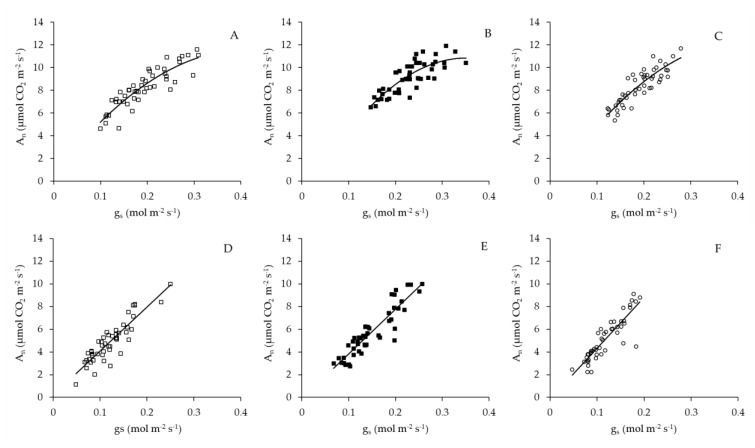
The response of photosynthesis rate (An) to stomatal conductance (*g*_s_) variations in ‘Fuji’ apple leaves grown in red (□), blue (■) and white control (○) nets measured under ambient light conditions before solar noon (**A**–**C**) and after solar noon (**D**–**F**), respectively.

Before and after solar noon a significant relationship (*p* < 0.01) was found between A_n_ and *g*_s_ for the leaves growing under colored and control nets (Figure 8; Table 5). At both times of the day, the variation in A_n_ of leaves growing under red and control nets is explained 80% and 77% by the variation in *g*_s_, respectively (Table 5). However, before solar noon the variation in A_n_ of leaves grown under blue net is only explained 67% by the variation in *g*_s_, while after noon solar the variation of A_n_ in leaves under blue net is 83% explained by a variation in *g*_s_ (Table 5). Before solar noon the relationship between A_n_ and *g*_s_ was better adjusted by a negative polynomial model (Figure 8A–C), whose slope was more negative and significant (β2 = −101.5; *p* < 0.05) in the blue net (Table 5). After midday the relationship between A_n_ and *g*_s_ was better adjusted to a positive linear model (Figure 8D–F), whose slope was statistically significant (*p* < 0.01) for the red, blue and control nets (Table 5).

## 3. Discussion

Trees under blue net showed a lower LMA compared with white (control) net. Decrease of LMA in response to reduction in light intensity have been widely documented in apple trees [23]. The differences in LMA result from alterations in thickness of leaf palisade tissue as well as in leaf area due mainly to light intensity changes [24]. Thus, leaves that develop under higher light availability show smaller area, greater thickness and more palisade layers when compared to those leaves developed under lower light availability [24]. Despite a comparable total leaf tissue thickness, microscopy analysis showed that palisade thickness and ratio palisade/spongy mesophyll under blue and red nets were decreased in 19% and 25%, respectively, compared to control (Figure 4; Table 3). These results demonstrate that the lowest LMA found under blue net was mainly due to decreases in thickness of palisade tissue and increases in the air space of mesophyll, which are normal adaptive responses of leaves grown under shading conditions to allow a better light transmission towards the chloroplasts [25]. Furthermore, these results demonstrate the plasticity of the mesophyll structure in apple leaves to modifications in light availability under netting [26]. Although the red net presented similar LMA to control, no significant changes were observed in mesophyll characteristics among leaves that develops under blue and red nets. Considering that PPFD availability was similarly reduced by red and blue nets (Figure 1A), these results suggest that, in our study, light quantity seemed to be more important than light quality for the modification of the anatomical characteristics of leaf palisade cell tissue as was also suggested by [27]. The differences found in stomatal frequency of leaves grown under colored nets confirm these results. Leaf stomata density did not differ among red and blue nets, but showed a reduction of 17% if compared to control (white net), similarly to [24], who found a reduction in leaf stomatal density when olives trees were exposed to reduced light intensity through continuous shading. However, the differences in leaf chlorophyll content between colored nets cannot be explained by changes in light intensity because the leaves grown under red and blue nets received the same quantity of PPFD (Figure 1A). The increase of chlorophyll content in leaves grown under Blue net has also been observed in ornamental plants [28]. Although the increase in chlorophyll content is also a common response of leaves grown to reduced light availability [1], we demonstrate that, regardless of PPFD intensity, chlorophyll content is a leaf characteristic stimulated specifically by blue net. Spectra light analysis demonstrated that blue net significantly increased the blue to red light ratio (Figure 2). Recent research have reported the importance of blue light on leaf chlorophyll enhancement in conditions with reduced visible and red light to allow a better photosynthetic capacity, the process of which is mediated by specific plant photoreceptors denominated phototropins [29].

The increases *g*_s_ under blue and red nets seem contradictory, because in leaves under these nets we found lower stomatal density, normally associated to less *g*_s_ [24]. In fruit trees, including apple, *g*_s_ is widely influenced by environmental and management factors affecting the plant water status [30]. The environmental conditions and agronomic management were the same in our experiment. Minimum values of leaf water potential (Figure 7) were up to −1.7 MPa, i.e., in accordance with values measured in well irrigated apple trees grown under field and pot conditions [31]. Moreover, there were no differences in leaf water potential among nets (Figure 7), therefore in our experiment the differences leaf *g*_s_ cannot be attributed to changes in plant water status. An alternative explanation could be associated to tree growth and development that were induced by colored nets. It has been reported that reduced shoot growth by dwarfing rootstocks negatively affected *g*_s_ in apple trees, while rootstocks inducing rapid shoot growth promote higher *g*_s_ [32]. Trees under blue net show larger total shoot length compared to red and control net (Table 1), which could be explained by phytochrome-mediated effect on shoot elongation [33] due to reduced R/FR ratio and the lowest phytochrome photo-equilibrium observed under blue net (Figure 1B). From this point of view, the greater *g*_s_ observed in leaves that develop under blue net can be explained by higher shoot growth, but they do not explain the effect of the red net on *g*_s_ improvement (Table 1). One further explanation can be related to the effect of colored nets on stomata dimensions. Scanning electron microscopy analysis demonstrated that leaves under blue and red nets (in particular Red) showed longer stomata and with greater length-to-width ratio compared to control net (Table 2). There is evidence that a reduction in stomatal density per surface area is negatively related to stomata size; thus, leaves with a lower stomatal density have greater stomata size and length. These morphological changes are induced as adaptation mechanisms to environmental conditions limiting stomatal conductance [34]. Furthermore, there is scientific evidence that “elongated” stomata type with a greater length/width ratio have the ability to perform gas exchange more quickly than the “kidney” type stomata with a lower length/width ratio [35]. Therefore, these arguments would explain why the leaves that grew under the blue and red nets presented a higher *g*_s_, despite a decrease in stomatal density due to lower light availability under these nets (Table 1 and Table 2).

Higher *g*_s_ observed under red and blue nets was also linked to increases in A_n_ and *E* (Table 4). These results are similar to those observed in other fruit species, where exposure of citrus trees to shading nets increased *g*_s_, *E* and A_n_ [15]. These results indicate that even though leaves under blue and red net develops as “shade leaves”, which could limit the photosynthetic machinery (lower palisade cell development and stomata density), this morphological changes did not affect their gas exchange performance, so more specific mechanisms must be involved in this response [29]. Leaf gas exchange measurements at ambient light conditions indicated that although red and blue reduced PPFD by the same amount compared to the control net (Figure 5A), leaf *g*_s_ was higher under blue than red net (Figure 5B). Differences in spectral light transmission (i.e., light quality) among colored nets are likely involved in these responses. Spectra analysis demonstrated that light composition under the Blue net was richer in blue photons proportion (400–500 nm), while reduced red light quantity (600–700 nm) was observed compared to the red net (Figure 1D; Figure 2). It has been widely documented that blue light wavelength is always more effective than red light in promoting stomata opening, as well as in preventing stomatal closure [18]. Therefore, the highest *g*_s_ found in leaves grown under blue net compared to red would be explained by a direct effect of blue light in promoting stomatal aperture [22]. Leaf *E* was always highest under Blue net (Figure 5D), which can be explained by stomatal control. Daily leaf *E* in apple trees is mainly affected by environmental conditions, thus high leaf *E* is usually related to high vapour pressure deficit, however beyond a certain threshold the relation between leaf *E* and vapour pressure deficit becomes non-linear due to feedback control of leaf *E* by *g*_s_ [30]. Therefore, the effect of blue net on stomatal opening was also reflected in significant increments in leaf *E*. Since A_n_ did not differ among colored nets, increased plant transpiration under blue net suggests a greater cost for the plant in water use. This was confirmed by the lowest value of IWUE observed under blue net (Figure 6D). Under environmental variations, the ability of crops to adjust their water use strategy is governed by non-stomatal (Rubisco activity and electron transport rate) and stomatal mechanisms. In this sense, the IWUE, defined as the relationship between A_n_ and *g*_s_, is less dependent on the variation in environmental conditions than the WUE, and it is generally controlled by genetic aspects in many species of fruit tree [36]. Considering that the instantaneous carboxylation efficiency did not vary significantly between netting (Figure 6B), this would indicate that the lower IWUE observed in the blue net should be regulated by the effect of the greater proportion of blue light that stimulates the opening of the stomata. From regression analysis it was possible to determine that, during the morning, a period with less availability of PPFD, the relationship between A_n_ and *g*_s_ showed a curvilinear and saturation behavior in leaves growing under red and blue nets. Indeed, the effect was more marked in leaves grown under blue net. However, at noon, with greater intensity of PPFD, the variation in A_n_ was linear and strongly dependent on variations in *g*_s_, with a greater effect under blue net. A curvilinear relationship would indicate the participation of a non-stomatal mechanism limiting photosynthesis, such as lower Rubisco activity or reduced electron transport rate [37]. In our study, no significant effect of the colored netting was found on the instantaneous carboxylation efficiency of Rubisco. Therefore, it is likely that it is rather an effect of the netting on lower development of leaf mesophyll cells (Figure 4; Table 3) and that they would be limiting the photosynthetic machinery under lower light conditions [24]. Finally, it has been shown that, in apple trees, cultivars in which a linear relationship between A_n_ and *g*_s_ is observed would show a greater stomatal limitation for photosynthesis and would have a greater water conserving strategy [36], indicating that red and blue nets differentially affect the stomatal regulation of leaf photosynthesis and transpiration of apple trees, whose responses depend on the intensity of solar radiation.

## 4. Materials and Methods

### 4.1. Plant Material and Experimental Design

The trial was carried out during 2009 and 2010 on two-year-old ‘Fuji’ apple trees grafted on dwarfing M9 rootstock and formed with feathers by the ‘knip-boom’ cultural practice [38]. Before the beginning of bud break, the weight, trunk diameter and height of 90 trees were quantified and the trees were divided in two homogeneous groups: Low-vigor and High-vigor. Trees were placed in 40 L pots (1:2 sand and soil mix) and randomly assigned to three N-S oriented rows at 2.5 × 1.0 m spacing, to avoid mutual shading between rows. Irrigation was supplied daily by a computer-controlled drip system. Mineral nutrition of trees was carried out weekly (from full bud break until shoot growth ceased) with 100 mL standard solution composed of nitrogen, potassium, phosphorus and microelements. Trees were managed by the following pruning and thinning practices, respectively: without removal of feathers and total removal of fruits.

**Figure 9:**
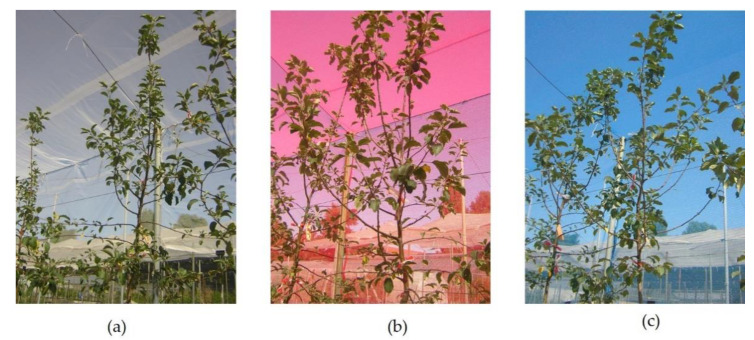
(**a**) Detail of ‘Fuji’ apple trees grown under white control, (**b**) red and (**c**) blue colored nets.

Blue and red colored shade nets with nominal shade factor 40% (ChromatiNet^®^, Polysack Industries, Negev, Israel) were installed over a metal tunnel arc 6 m wide and 3.5 m high. East and West sides of the tunnel were covered to the ground, while the North and South ends of the tunnel were left uncovered to insure good air circulation. Due to frequent hail storms, a white neutral net at 20% shading was incorporated as control to examine the effect of light quantity in addition to evaluate the effect of light quality (red and blue light composition) by colored nets (Figure 9). Nets were installed when blooming was finished (April) and removed at leaf fall (November). The experiment was arranged in a completely randomized strip block design on plots composed of 18 potted trees each and three replications.

### 4.2. Light Relations

The influence of nets on light quantity was measured as changes in photosynthetic photon flux density (PPFD, µmol m^−2^ s^−1^) using a QSO-S quantum sensor (Decagon Devices, Pullman, WA, USA), whereas the effect of nets on light quality was estimated as variation in spectral light composition using an optical fiber with a diffuser installed on the measuring head and connected to the LI-1800 spectroradiometer (LI-COR, Lincoln, NE, USA). The PPFD incident and spectral light composition were simultaneously measured at 1 m above ground in the alleyway among the potted trees. Readings were carried out under a sunny day and replicated in four points under each color net and three times during the day (mid-morning, solar noon and mid-afternoon). Phytochrome and cryptochrome light-related parameters were estimated by red (600–700 nm) to far-red (700–800 nm) ratio (R/FR) and blue (400–500 nm) to red (600–700 nm) ratio (B/R), respectively, according to [39]. The phytochrome photoequilibrium (Φ_c_) was mathematically estimated by the model described by [40].

### 4.3. Tree Growth

Total shoot length, number of shoots and trunk diameter (d) above the scion-rootstock union were measured over a total of trees per color net. Trunk-cross section area (TCSA, cm2) was calculated by the function п (d/2)^2^.

### 4.4. Leaf Morphology and Anatomy

Six fully expanded sun-exposed leaves per colored net were randomly collected from the middle part of 1-year shoots on 29 July 2009. Leaves were put in plastic bags into a cooler box with dry-ice and carried to the laboratory for further analysis. After removing the trichomes with adhesive tape, the stomatal density was determined by epidermal impressions with transparent nail polish [41]. Nail polish was placed in two leaf sections over the abaxial surface separated by the central vein. Once dried, the nail polish film was gently removed and mounted on microscope glass slides with distilled water. Stomata number was counted from digital image recorded on two square areas of 0.25 mm^2^ per leaf section by a charge-coupled device (CCD) camera mounted on a light microscope at 40× magnification (Leitz DM RB, Leitz, Wetzlar, Germany).

Length of stomata was analyzed by the scanning electron microscopy (SEM) procedure [42]. Four leaves per color net were collected and peeled by adhesive tape. Then, a small piece (~50 mm^2^) cut from the middle of the lamina of each leaves were fixed in a formalin-free fixative (FineFix, Milstone, Bergamo, Italy), dehydrated at different ethanol increasing levels and desiccated with a critical point dryer (CPD) processor (Balzers CPD 030, Schalksmühlen, Germany). Leaf samples with the abaxial side up were mounted on aluminum stubs and gold-coated with an sputter coater device (SCD) (Balzers, Liechtenstein, Germany). Microscopical analysis was made with a SEM 515 (Philips, Amsterdam, Netherlands) at 20–25 kV. Four images in different points for each sample were taken. Length of each stomata was measured as the distance between the outside edges and then all stomata were classified in four categories: <15 µm, 15–20 µm, 20–25 µm, and >25 µm.

Leaf mesophyll analysis was carried out through histological procedures [42]. Four well-illuminated leaves per color net were collected from the middle part of annual shoots. Leaf tissue samples (~60 mm^2^) were cut from the middle of the lamina. Samples were fixed in a formalin-free fixative (FineFix, Milstone, Bergamo, Italy), dehydrated in ethanol series (50%, 70%, 80%, and 90%) and gradually embedded in glycol methacrylate (Technovit 7100; Heraeus Kulzer GmbH, Werheim, Germany). Twenty cross-sections 3 µm thick were taken per each leaf sample through a rotary microtome (Reichert-Jung, Heidelberg, Germany). Then, the sample sections were mounted on glass slides with distilled water and observed by light microscope at 10× magnification to select those with appropriate morphological definition. Selected sections were stained with toluidine blue for 5 min and photographed by a CCD camera connected to the light microscope at 40× magnification (Leitz DM RB, Leitz, Wetzlar, Germany). Thickness (µm) of leaf blade, upper and lower epidermis, palisade and spongy mesophyll were measured in at least four different sections per each leaf and sample. Palisade to spongy mesophyll ratio was calculated [43]. All measurements (stomata and mesophyll structure) were processed with Aequitas image analysis software program.

Leaf chlorophyll content was measured using a SPAD 502 chlorophyll meter (Konica Minolta Sensing Inc., Osaka, Japan) in six well-illuminated leaves from the middle part of one-year shoots. Subsequently, additional mature and fully six exposed leaves were randomly collected and the single leaf area (cm^2^) was determined using a LI-3000 area meter (LI-COR, Lincoln, NE, USA). Then, leaves were dried in a forced drought oven at 60° C until reaching a constant weight and leaf dry mass per area unit (LMA, mg cm^−2^) was estimated.

### 4.5. Leaf Gas Exchange

Net photosynthetic rate (A_n_, µmol CO_2_ m^−2^s^−1^), stomatal conductance (*g*_s_, mol m^−2^s^−1^), transpiration rate (*E*, mmol H_2_O m^−2^s^−1^) and intercellular carbon dioxide concentration (C_i_, µmol mol^−1^) were measured using a LI-6400 gas infrared analyzer (LI-COR, Lincoln, NE, USA) at controlled and saturating photosynthetic photon flux (1200 µmol m^−2^s^−1^) provided by internal red/blue light-emitting diode (LED) light. Leaf gas exchange measurements were carried out during a sunny day (3 July 2009) in the morning (10:00–11:30 h) when maximum *g*_s_ are observed in apple trees [30], and within the same period in which leaf samples for morphological analysis were taken.

During 2010, the daily course of A_n_, *g*_s_, C_i_, *E* and PPFD were measured through a LI-6400 infrared gas analyzer (LI-COR, Lincoln, NE, USA). Gas exchange parameters were taken on six sun exposed and fully expanded leaves selected from the middle part of one-year shoots. All measurements were made at 1 h intervals (from 8:30 a.m. to 16:15 p.m.), on a sunny day with direct sunlight conditions. Water use efficiency (WUE) as the ratio of A_n_/*E* (µmol CO_2_ mmol^−1^ H_2_O), intrinsic water use efficiency (IWUE) as the ratio of A_n_/*g*_s_ (µmol CO_2_ mol^−1^ H_2_O) and instantaneous carboxylation efficiency as ratio of A_n_/C_i_ were computed.

### 4.6. Tree Water Status

Complementary, predawn and daily course (4:00 a.m.–22:00 p.m.) of leaf water potential (MPa) were estimated by a Scholander-type pressure chamber (PMS Instruments, Corvallis, OR, USA). Measurements were taken during a partially sunny day (17 August 2010), on five mature and fully exposed leaves from the same positions as those used for gas exchange analysis.

### 4.7. Statistical Analysis

One and two-way analysis of variance (ANOVA) at 0.05 of significance was performed to detect statistical differences among treatments, followed by the LSD Fischer test for comparison of mean values. Linear regression analysis was carried out to model leaf photosynthesis variation in response to stomatal conductance. Statistical analysis was performed by the Infostat and R environment software [44].

## 5. Conclusions

Red and blue nets alter in a similar way (reduction of PPFD intensity) leaf morpho-anatomical traits without negative effects on photosynthesis in apple trees.

Irrespective of PPFD intensity, blue and red nets alter in different ways (changes in red and blue light proportions) the stomatal regulation of leaf photosynthesis and transpiration in apple trees.

These results provide a new insight on the potential use of red and blue nets for differential modulating of leaf gas exchange in apple trees under orchard conditions and through the intelligent management of sunlight.

## Figures and Tables

**Table 1:** The influence of red and blue netting and tree vigor on leaf area (LA), leaf mass area (LMA), leaf stomata density (SD), leaf chlorophyll content (ChC), trunk cross section area (TCSA) and total shoot length (TSL) in ‘Fuji’ apple trees.

Factors	LA (cm^2^)	LMA (mg cm^−2^)	SD (n° mm^−2^)	ChC (SPAD Unit)	TCSA (cm^−2^)	TSL (m)
Net system (NS)						
Control	16.4	12.8 a ^1^	531.8 a	48.5 b	4.8	3.3 b
Red	20.6	12.3 ab	442.2 b	47.9 b	4.6	4.2 b
Blue	24.9	11.8 b	438.7 b	50.9 a	4.6	5.6 a
*p-*value	0.0640 ^ns^	0.0286 *	0.0343 *	<0.0001 **	0.8164 ^ns^	0.0129 *
Tree vigor (TV)						
High	21.9	12.3	478.4	49.4	4.8	4.2
Low	21.5	12.3	463.3	48.8	4.5	4.4
*p-*value	0.8161 ^ns^	0.8798 ^ns^	0.6331 ^ns^	0.0945 ^ns^	0.3361 ^ns^	0.7030 ^ns^
*p-*value NS × TV	0.7462 ^ns^	0.6693 ^ns^	0.5154 ^ns^	0.3903 ^ns^	0.4059 ^ns^	0.4886 ^ns^

^1^ Mean separation within rows by the LSD Fischer test; *n* = 6 leaves. *; **; ^ns^: Significant, highly significant and non-significant at *p* < 0.05 and 0.01, respectively.

**Table 2:** The influence of Red and Blue netting on stomata length (SL), stomata width (SW), length to width (L/W) ratio and stomata frequency in ‘Fuji’ apple leaves.

Net Systems	SL (µm)	SW (µm)	L/W	Stomata Frequency by Length (%)			
				<15 µm	15–20 µm	20–25 µm	>25 µm
Control	20.2 b	14.5	1.4 b	5.0	45.0	40.0 b	10.0
Red	22.5 a	14.6	1.5 a	1.0	26.0	51.0 a	22.0
Blue	21.3 ab	13.8	1.5 a	1.0	32.0	56.0 a	11.0
*p-*value	0.0262 *	0.3531 ^ns^	0.0029 **	0.0537 ^ns^	0.0667 ^ns^	0.0193 *	0.1104 ^ns^

*n* = 4 leaves. *; **; ^ns^: Significant, highly significant and non-significant at *p* < 0.05 and 0.01, respectively.

**Table 3:** The influence of red and blue netting on leaf mesophyll characteristics in ‘Fuji’ apple trees.

Net System	Leaf Tissues Thickness (µm)				Palisade/Spongy Mesophyll Ratio
	Total	Upper Epidermis	Lower Epidermis	Palisade	
Control	228.4	13.7	9.7	116.2 a ^1^	1.4 a
Red	218.9	13.4	9.7	96.2 b	1.0 b
Blue	215.2	13.6	9.8	99.0 b	1.1 b
*p-*value	0.4980 ^ns^	0.9123 ^ns^	0.9616 ^ns^	0.0404 *	0.0015 **

^1^ Mean separation within rows by the LSD Fischer test; *n* = 4 leaves. *; **; ^ns^: Significant, highly significant and non-significant at *p* < 0.05 and 0.01, respectively.

**Table 4:** The influence of red and blue netting and tree vigor on net photosynthesis rate (*A_n_*), stomatal conductance (*g*_s_), transpiration rate (*E*) and intercellular carbon dioxide concentration (C_i_) in ‘Fuji’ apple leaves measured under controlled light conditions.

Factors	A_n_ (µmol CO_2_ m^−2^ s^−1^)	*g*_s_ (mol m^−2^ s^−1^)	*E* (mmol H_2_O m^−2^ s^−1^)	C_i_ (µmol mol^−1^)
Net system (NS)				
Control	11.2 c ^1^	0.16 b	4.0 b	248.0
Red	12.9 b	0.23 a	5.2 a	265.4
Blue	14.7 a	0.23 a	5.2 a	253.7
*p-*value	0.0037 **	0.0090 **	0.0199 *	0.1158 ^ns^
Tree Vigor (TV)				
High	13.2	0.19	4.8	246.3
Low	12.7	0.22	4.8	265.1
*p-*value	0.7369 ^ns^	0.2670 ^ns^	0.9112 ^ns^	0.0565 ^ns^
*p-*value NS × TV	0.6004 ^ns^	0.6720 ^ns^	0.4490 ^ns^	0.6075 ^ns^

^1^ Mean separation within rows by the LSD Fischer test; *n* = 6 leaves. *; **; ^ns^: Significant, highly significant and non-significant at *p* < 0.05 and 0.01, respectively.

**Table 5:** Regression coefficients for the relationship between photosynthesis rate and stomatal conductance in ‘Fuji’ apple leaves as affected by red, blue and control nets before and after solar noon.

Regression Coefficients	Net Systems		
	Control	Red	Blue
	Before Solar Noon		
β_0_	−0.5 ^ns^	0.49 ^ns^	−1.6 ^ns^
β_1_	60.4 **	53.6 **	71.3 **
β_2_	−70.4 ^ns^	−64.8 ^ns^	−101.5 *
R_2_	0.77 **	0.80 **	0.67 **
	After Solar Noon		
β_0_	−0.15 ^ns^	0.19 ^ns^	−0.13 ^ns^
β_1_	44.8 **	38.8 **	39.4 **
R_2_	0.77 **	0.80 **	0.83 **

Regression equation are Y = β_0_ + β_1_X + β_2_X^2^ (before solar noon) and Y = β_0_ + β_1_X (after solar noon). *; **; ^ns^: Significantly, highly significantly and non-significantly at *p* < 0.05 and 0.01, respectively.

## Data Availability

The data presented in this study are available on request from the corresponding author.

## References

[1] Solomakhin A., Blanke M.M. (2008). Coloured hailnets alter light transmission, spectra and phytochrome, as well as vegetative growth, leaf chlorophyll and photosynthesis and reduce flower induction of apple. Plant. Growth Regul..

[2] Gindaba J., Wand S.J.E. (2007). Do fruit sunburn control measures affect leaf photosynthetic rate and stomatal conductance in ‘Royal Gala’ apple?. Environ. Exp. Bot..

[3] Mupambi G., Anthony B.M., Layne D.R., Musacchi S., Serra S., Schmidt T., Kalcsits L.A. (2018). The influence of protective netting on tree physiology and fruit quality of apple: A review. Sci. Hortic..

[4] Olivares-Soto H., Bastías R.M. (2018). Photosynthetic efficiency of apples under protected shade nets. Chil. J. Agric. Res..

[5] Chouinard G., Veilleux J., Pelletier F., Larose M., Philion V., Joubert V., Cormier D. (2019). Impact of Exclusion Netting Row Covers on ‘Honeycrisp’ Apple Trees Grown under Northeastern North American Conditions: Effects on Photosynthesis and Fruit Quality. Insects.

[6] Kalcsits L., Musacchi S., Layne D.R., Schmidt T., Mupambia G., Serra S., Mendozac M., Asteggiano L., Jarolmasjed S., Sankar S. (2017). Above and below-ground environmental changes associated with the use of photoselective protective netting to reduce sunburn in apple. Agric. For. Meteorol..

[7] Shahak Y., Ratner K., Giller Y., Zur N., Or E., Gussakovsky E., Stern R., Sarig P., Raban E., Harcavi E. (2008). Improving solar energy utilization, productivity and fruit quality in orchards and vineyeards by photoselective netting. Act. Hortic..

[8] Lobos G.A., Retamales J.B., Hancock J.F., Flore J.A., Romero-Bravo S., Del Pozo A. (2013). Productivity and fruit quality of Vaccinium corymbosum cv. Elliott under photo-selective shading nets. Sci. Hortic..

[9] Basile B., Giaccone M., Cirillo C., Ritieni A., Graziani G., Shahak Y., Forlania M. (2012). Photo-selective hail nets affect fruit size and quality in Hayward kiwifruit. Sci. Hortic..

[10] Zhou K., Jerszurki D., Avi Sadk A., Shlizerman L., Rachmilevitch S., Ephrath J. (2018). Effects of photoselective netting on root growth and development of young grafted orange trees under semi-arid climate. Sci. Hortic..

[11] Bastías R.M., Manfrini L., Corelli-Grappadelli L. (2012). Exploring the potential use of photoselective nets for fruit growth regulation in apple. Chil. J. Agric. Res..

[12] Corollaro M.L., Manfrini L., Endrizzi I., Aprea E., Demattè M.L., Charles M., Bergamaschi M., Biasioli F., Zibordi M., Corelli Grappadelli L. (2015). The effect of two orchard light management practices on the sensory quality of apple: Fruit thinning by shading or photo-selective nets. J. Hort. Sci. Biotech..

[13] Olivares-Soto H., Bastías R.M., Calderón-Orellana A., López M.D. (2020). Sunburn control by nets differentially affects the antioxidant properties of fruit peel in ‘Gala’ and ‘Fuji’ apples. Hortic. Environ. Biotechnol..

[14] Lobos G.A., Retamales J.B., Hancock J.F., Flore J.A., Cobo N., Del Pozo A. (2012). Spectral irradiance, gas exchange characteristics and leaf traits of *Vaccinium corymbosum* L. ‘Elliott’ grown under photo-selective nets. Environ. Exp. Bot..

[15] Medina C.L., Souza R.P., Machado E.C., Ribeiro R.V., Silva J.A.B. (2002). Photosynthetic response of citrus grown under reflective aluminized polypropylene shading nets. Sci. Hortic..

[16] Mupambi G., Musacchi S., Serra S., Kalcsits L.A., Layne D.R., Schmidt T. (2018). Protective netting improves leaf-level photosynthetic light use efficiency in ‘Honeycrisp’ apple under heat stress. HortScience.

[17] Bastías R.M., Corelli-Grappadelli L. (2012). Light quality management in fruit orchards: Physiological and technological aspects. Chil. J. Agric. Res..

[18] Zheng L., Van Labeke M.C. (2017). Long-Term effects of red- and blue-light emitting diodes on leaf anatomy and photosynthetic efficiency of three ornamental pot plants. Front. Plant. Sci..

[19] Baraldi R., Rapparini F., Rotondi A., Bertazza G. (1998). Effects of simulated light environments on growth and leaf morphology of peach plants. J. Hort. Sci. Biotech..

[20] Schuerger A.C., Brown C.S., Stryjewski E.C. (1997). Anatomical features of pepper plants (*Capsicum annuum* L.) Grown under red light-emitting diodes supplemented with blue or far-red light. Ann. Bot..

[21] Wang H., Gu M., Cui J., Shi K., Zhou Y., Yu J. (2009). Effects of light quality on CO2 assimilation, chlorophyll-fluorescence quenching, expression of Calvin cycle genes and carbohydrate accumulation in *Cucumis sativus*. J. Photochem. Photobiol..

[22] Shimazaki K., Doi M., Assmann S.M., Kinoshita T. (2007). Light regulation of stomatal movement. Annu. Rev. Plant. Biol..

[23] Corelli-Grappadelli L., Ferree D.C., Warrington I.J. (2003). Light Relations. Apples: Botany, Production and Uses.

[24] Gregoriou K., Pontikis K., Vemmos S. (2007). Effects of reduced irradiance on leaf morphology, photosynthetic capacity, and fruit yield in olive (*Olea europea* L.). Photosynthetica.

[25] Smith K.W., Vogelmann T.C., De Lucia E., Bell D.T., Shepherd K.A. (1997). Leaf form and photosynthesis. Do leaf structure and orientation interact to regulate light and carbon dioxide?. Bioscience.

[26] Solomakhin A., Blanke M. (2010). The microclimate under coloured hailnets affects leaf and fruit temperature, leaf anatomy, vegetative and reproductive growth as well as fruit colouration in apple. Ann. App. Biol..

[27] Kim G.-T., Yano S., Kozuka T., Tsukaya H. (2005). Photomorphogenesis of leaves: Shade avoidance and differentiation of sun and shade leaves. Photochem. Photobiol. Sci..

[28] Stamps R.H., Chandler A.L. (2008). Differential effect of colored shade nets on three cut foliage crops. Act. Hortic..

[29] Takemiya A., Inue S.I., Doi M., Kinoshita T., Shimazaki K.I. (2005). Phototropins promotes plant growth in Responses to Blue Light in Low Light Environments. Plant. Cell..

[30] Giuliani R., Nerozzi F., Magnanini E., Corelli-Grappadelli L. (1997). Influence of environmental and plant factors on canopy photosynthesis and transpiration in apple trees. Tree Physiol..

[31] Lakso A.N., Ferree D.C., Warrington I.J. (2003). 2003. Water Relations of Apples. Apples: Botany, Production and Uses.

[32] Atkinson C.J., Policarpo M., Webster A.D., Kingswell G. (2000). Drought tolerance of clonal Malus determined from measurements of stomatal conductance and leaf water potential. Tree Physiol..

[33] Smith H. (2000). Phytochromes and light signal perception by plants–an emerging synthesis. Nature.

[34] Franks P., Drake P.L., Berling D.J. (2009). Plasticity in maximum stomatal conductance constrained by negative correlation between stomatal size and density: An analysis using Eucalyptus globulus. Plant. Cell Environ..

[35] Franks P., Farquhar G.D. (2007). The mechanical diversity of stomata and its significance in gas-exchange control. Plant. Physiol..

[36] Massonnet C., Costes E., Rambal S., Dreyer E., Regnard J.L. (2007). Stomatal regulation of photosynthesis in apple leaves: Evidence for different water-use strategies between two cultivars. Ann. Bot..

[37] Farquhar G.D., Sharkey T.D. (1982). Stomatal conductance and photosynthesis. Ann. Rev. Plant. Physiol..

[38] Wilton J. (2001). Apple production trends in Europe. Compact Fruit Tree..

[39] Kittas C., Baille A., Giaglaras P. (1999). Influence of covering material and shading on the spectral distribution of light in greenhouses. J. Agric. Eng. Res..

[40] Sager J.C., Smith W.O., Edwards J.L., Cyr K.L. (1988). Photosynthetic efficiency and phytochrome photoequlibria determination using spectral data. Trans. Am. Soc. Agric. Eng..

[41] Gitz D., Baker J. (2009). Methods for creating stomatal impressions directly onto archivable slides. Agron. J..

[42] Chieco C., Rotondi A., Morrone L., Rapparini F., Baraldi R. (2013). An ethanol-based fixation method for anatomical and micro-morphological characterization of leaves of various tree species. Biotech. Histochem..

[43] Baldini E., Facini O., Nerozzi F., Rossi F., Rotondi A. (1997). Leaf characteristics and optical properties of different woody species. Trees Struct. Fun.

[44] Balzarini M.G., González L., Tablada M., Casanoves F., Di Rienzo J.A., Robledo C.W. (2008). InfoStat: Statistical Software.

